# Two-Step Real-Time Night-Time Fire Detection in an Urban Environment Using Static ELASTIC-YOLOv3 and Temporal Fire-Tube

**DOI:** 10.3390/s20082202

**Published:** 2020-04-13

**Authors:** MinJi Park, Byoung Chul Ko

**Affiliations:** Department of Computer Engineering, Keimyung University, Daegu 42601, Korea; anmj8780@stu.kmu.ac.kr

**Keywords:** night fire detection, EASTIC-YOLOv3, bag-of-features, fire-tube, random forest

## Abstract

While the number of casualties and amount of property damage caused by fires in urban areas are increasing each year, studies on their automatic detection have not maintained pace with the scale of such fire damage. Camera-based fire detection systems have numerous advantages over conventional sensor-based methods, but most research in this area has been limited to daytime use. However, night-time fire detection in urban areas is more difficult to achieve than daytime detection owing to the presence of ambient lighting such as headlights, neon signs, and streetlights. Therefore, in this study, we propose an algorithm that can quickly detect a fire at night in urban areas by reflecting its night-time characteristics. It is termed ELASTIC-YOLOv3 (which is an improvement over the existing YOLOv3) to detect fire candidate areas quickly and accurately, regardless of the size of the fire during the pre-processing stage. To reflect the dynamic characteristics of a night-time flame, N frames are accumulated to create a temporal fire-tube, and a histogram of the optical flow of the flame is extracted from the fire-tube and converted into a bag-of-features (BoF) histogram. The BoF is then applied to a random forest classifier, which achieves a fast classification and high classification performance of the tabular features to verify a fire candidate. Based on a performance comparison against a few other state-of-the-art fire detection methods, the proposed method can increase the fire detection at night compared to deep neural network (DNN)-based methods and achieves a reduced processing time without any loss in accuracy.

## 1. Introduction

Among the various types of disasters, fires are often caused by human carelessness and can be prevented sufficiently in advance. Fires can be categorized as occurring under natural conditions, such as forest fires, and in urban areas such as buildings and public places. Forest fire detection is very different from indoor or short-range fire detection due to atmospheric conditions (clouds, shadows, and dust particle formation), slow spreading, ambiguous shapes, and color patterns [1,2]. Therefore, fire detection in urban areas or indoors requires different approaches from forest fire.

With the rapid urbanization and an increase in the number of high-rise buildings, fires in urban areas are more dangerous than forest fires in terms of human casualties and property damage. According to a report by the Korea National Fire Agency, 28,013 fires occurred in buildings and structures in 2019, accounting for 66.2% of all fires that year. These building fires resulted in 316 deaths, 1915 injuries and $415 million in damage, which are the greatest numbers incurred annually in recent years [3]. Considering that industrial society is continuously growing, this phenomenon will continue for the time being. In particular, fires in buildings or public places are more likely to occur at night when people are less active. Therefore, improvements in the accuracy of night-time fire detection can greatly reduce property damage from fires.

Conventional fire alarm systems using optical, infrared, and ion sensors are not triggered until smoke, heat or radiation actually reach the sensor and typically cannot provide additional information such as the fire location, size or degree of combustion. Therefore, when an alarm is triggered, the system manager should visit that location to see if a fire is present [4].

Camera-sensor-based fire detection systems have recently been proposed to overcome the limitations of existing sensors. Unlike existing sensor-based fire detection approaches, the advantages of vision-based fire detection systems can be summarized as follows [5]: (1) the cost for the equipment is relatively lower than that of optical, infrared, and ion sensors; (2) the response time for fire and smoke detection is faster; (3) the system functions as a volume sensor and can therefore monitor a large area; and (4) the system manager can confirm the existence of the fire without visiting the location. Unlike a normal visible camera, a fire may be detected using a thermal camera. Although the potential flame area can be easily detected using a thermal camera by measuring the heat energy emitted from the fire, detecting fires using this method has several problems in urban areas, unlike natural environments such as mountains and fields [6]. First, a thermal camera is more expensive than a normal visible camera, and surrounding objects such as automobile engines, streetlights and neon signs can have heat energy similar to that of a fire flame when the thermal camera is far away. In addition, the difference in thermal energy between hot summer and cold winter occurs due to radiant heat on the surface [7], so the fire classification model training must be made differently.

**Figure 1:**
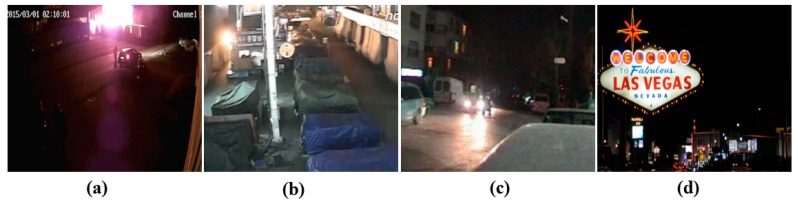
Examples of night-time fire and night-time fire-like lights: (**a**) a constantly changing size and shape of the fire without color information, (**b**) fire occurring in the corner of the building, (**c**) fire-like streetlight and car headlight, and (**d**) fire-like neon sign.

Conventional camera-based fire detection methods have applied algorithms developed based on the basic assumption that fire flames have a reddish color with a continuous upward motion. Therefore, classic camera-based methods use the color or differences in the information within the frame as a pre-processing step [4,5,8,9]. However, because the physical characteristics between fires at day and night are extremely different, a false detection may occur if a daytime fire detection algorithm is applied to a fire at night without a proper modification. Night-time fires located at a medium to long distance from the camera have the following differences compared to daytime fires, as shown in Figure 1a,b:

- a loss of color information;
- a relatively high brightness value compared to the surroundings;
- various changes in the shape and size from light blurring; and
- movements of the flames in all directions (daytime flames tend to move in an upward direction).

By contrast, fire-like lights such as neon signs, streetlights and the headlights of vehicles have a similar brightness, shape and reflection as real night-time fires, as shown in Figure 1c,d.

In conventional fire flame detection, the candidate flame regions are initially detected using a background subtraction method, whereas non-flame colored blocks are filtered out using color models and some conditional rules. These processes are essential steps for verification. Various parameters can then be used to characterize a flame for classification, such as the color, texture, motion and shape. After feature extraction, the learning of the pattern classifiers such as finite automata [4], fuzzy logic [5,10], support vector machine (SVM) [11], the Bayesian algorithm [12,13], neural network [14] and random forest [15] is conducted based on the feature vectors of the training data. Finally, the candidate flame regions are classified into fire- or non-fire flame regions using the pattern classifiers.

By contrast, deep learning-based fire detection has recently exhibited state-of-the-art performance in fire detection tasks. This approach significantly reduces the dependency of hand-crafted feature extraction and other pre-processing procedures through end-to-end learning that occurs directly in the pipeline from the input images. In particular, the application of a convolutional neural network (CNN) [16] significantly improves the performance of conventional machine learning-based algorithms for image-based fire detection. In a CNN-based approach, the input image is transformed through a collection of filters in the convolutional layer to create a feature map. Each feature map is then combined into a fully connected network, and the fire is recognized as belonging to a specific class based on the output of the softmax algorithm.

This paper focuses on fire detection using a camera, particularly for night-time fires, which are more difficult to detect than daytime fires. Owing to the different characteristics of daytime and night-time fires, a specialized algorithm for night-time fires is required. The application of a CNN to detect fire regions in still images can be a very useful approach. However, as mentioned above, night-time fires have similar characteristics as vehicle headlights or neon signs, and we therefore cannot achieve a high fire detection accuracy when a CNN is used alone. An additional procedure is needed to distinguish between real fire regions and regions with fire-like characteristics at night. As an alternative, an efficient approach is to combine a recurrent neural network (RNN) [17] or long short-term memory (LSTM) [18] with a CNN instead of using a CNN alone to consider the continual movement of a flame. However, this approach requires more computations, making real-time processing difficult to achieve.

## 2. Related Studies

Fire detection studies range from traditional sensor-based methods to the latest camera-based methods. Among them, this paper focuses on camera-based approaches, which have been actively studied in recent years. Camera-based fire approaches can be divided into machine learning and CNN-based methods, which are popular deep learning models. This section introduces some representative algorithms used in three different approaches and analyses the advantages and disadvantages of each.

### 2.1. Machine Learning and Deep Learning-Based Fire Detection

Chen et al. [19] proposed a fire detection algorithm using an RGB color model. However, the fire color depends on the burning material, and thus, additional features are required for accurate fire detection. To solve these problems, Töreyin et al. [20] used a spatial wavelet transform and a temporal wavelet transform to determine the presence of a fire, as well as the color change of the moving region. Ko et al. [11] used a two-class SVM classifier with a radial basis function kernel based on a temporal fire model with wavelet coefficients. Apart from the SVM, fuzzy logic was successfully applied to various fire videos using probability density membership functions based on a variation in the intensity, wavelet energy, and motion orientation on the time axis [21]. Ko et al. [5] proposed fuzzy logic with Gaussian membership functions of the fire shape, size, and motion variation to verify the fire region using a stereo camera.

Because analyzing the dynamic movement of a fire flame is an important factor in improving the fire detection performance, Dimitropoulos et al. [22] used an SVM classifier and the spatio-temporal consistency energy of each candidate fire region by exploiting prior knowledge regarding the possible existence of a fire in the neighboring blocks based on the current and previous frames. In addition, Foggia et al. [9] proposed a multi-expert system combining complementary feature information based on the color, shape variation and a motion analysis.

In the conventional approaches mentioned above, the features and classifiers for fire detection are generally determined by an expert. Many known hand-crafted features such as color, texture, shape and motion are used as the input features, and fire detection is based on pre-trained classifiers such as an SVM, fuzzy logic, AdaBoost, and random forest. Conventional approaches require relatively lower computing power and memory than deep learning-based approaches, but they achieve a relatively poor fire detection performance in a variety of environments. To solve this problem, CNN-based methods have recently been applied to enable end-to-end learning and minimize the amount of expert interference in feature extraction and classifier decisions after applying a basic model design.

Deep neural network (DNN)-based fire detection studies have recently been actively carried out. Such studies can be divided into the use of a CNN using still images and an RNN (or LSTM) with sequence images.

Frizzi et al. [23] used a simple CNN model having the ability to apply feature extraction and classification within the same architecture. This approach was proven to achieve a better classification performance than some other relevant conventional fire detection methods. Zhang et al. [24] proposed a joined deep CNN. With this method, the fire detection is applied in a cascaded fashion; thus, the full image is first tested using the global image-level classifier, and if a fire is detected, the fine-grained patch classifier is followed to detect the precise location of the fire patches.

Muhammad et al. [25] used a lightweight CNN based on the SqueezeNet [26] architecture for fire detection, localization, and semantic understanding of a fire scene. Muhammad et al. [27] also replaced SqueezeNet with GoogleNet [28] for fire detection using a CCTV surveillance network. Dunning and Breckon [29] investigated the automatic segmentation of fire pixel regions (super-pixel) in an image within the real-time bounds without reliance on the temporal scene information. After the clustering of a fire image, a GoogleNet [28] or simpler network is applied to classify the super-pixels of a real fire. In addition, Barmpoutis et al. [30] proposed a fire detection method in which Faster R-CNN [31] was applied followed by an analysis of the multi-dimensional textures in the candidate fire regions.

Most CNN-based approaches use a deep or shallow CNN network to confirm a fire region or detect a fire candidate as the first step and then apply additional techniques to verify the fire candidates. However, because fire detection is applied based on a CNN with a still image without considering the temporal variation of the flame, such methods have a high probability of a false detection of the surrounding fire-like objects as an actual fire. To solve this problem, some methods [32,33,34] have combined an RNN or an LSTM with a CNN when considering the spatio-temporal characteristics of the sequential fire flames. With these approaches, a CNN is normally used to extract the spatial features, and an RNN or LSTM is used to learn the temporal relation between frames. Han et al. [32] combined a CNN with an RNN in a consecutive manner such that the sequence data could be allowed in the model. Cao et al. [33] proposed an attention-enhanced bidirectional LSTM for camera-based forest fire recognition. Kim and Lee [34] used Faster R-CNN to detect the suspected regions of a fire and then summarized the features within the bounding boxes in successive frames accumulated by the LSTM to classify whether a fire was present within a short-term period.

Although these LSTM- or RNN-based approaches have a lower false detection ratio than a CNN-only fire detection method, it remains difficult to model the irregular moving patterns of a fire flame because only limited frames can be used simultaneously owing to the memory capacity. In addition, because a CNN and the LSTM layers must be applied in each frame, a large number of parameters and operations make real-time processing difficult to achieve.

Most of the existing studies related to fire detection have thus far been aimed at a daytime environment. In such an environment, flames have prominent colors, shapes and movements, providing more reliable results. However, in night-time environments, the movements of the flames are relatively small, making it difficult to distinguish from the surrounding lights that have similar characteristics, such as car headlights, streetlights, and neon signs. In particular, daytime fires can be easily detected by people, allowing for an early evolution, whereas fires at night occur when there are no people in the building or when people are asleep. Therefore, night-time fires are difficult to detect early, and if a fire breaks out, the loss of life and property becomes much greater than those of a daytime fire. Therefore, a fire monitoring algorithm specialized for night-time fires is needed.

### 2.2. Contributions of this Work

Unlike related approaches that use only a CNN or a CNN with an RNN (LSTM), a CNN is combined with an RF (which is a representative algorithm of an ensemble model) in this study, to improve the speed and accuracy of the fire detection. In addition, fire-tubes are generated by combining successive fire candidate areas for fire verification instead of an LSTM, and the accuracy of the fire detection is improved by learning irregular and dynamic fire motion patterns. In particular, the focus of this study is on night-time fire detection in urban areas, which is relatively more difficult to achieve than daytime fire detection. The results on various types of video show the excellent performance of the proposed approach.

In a previous study of Park et al. [35], we introduced a simple model of a night-time fire detection system using a fire-tube and RF classifier based on temporal information. However, for more accurate fire detection, we improved the basic ELASTIC model structure to increase the accuracy on small sized fires. Second, using the previous method, a linear fire-tube was generated in previous successive frames based on the fire area of the current frame, whereas in the current approach, we redesign a nonlinear fire-tube by extracting the regions as closely as possible to the actual fire region in every frame. Third, a codebook is constructed by applying more training data, and an RF is regenerated by using more various night-time fire training videos. Moreover, we prove the successful performance of the proposed method using more night-time fire datasets and confirm that the detection accuracy of a night-time fire is higher than that of other related CNN- and LSTM-based methods with a shorter processing time.

**Figure 2:**
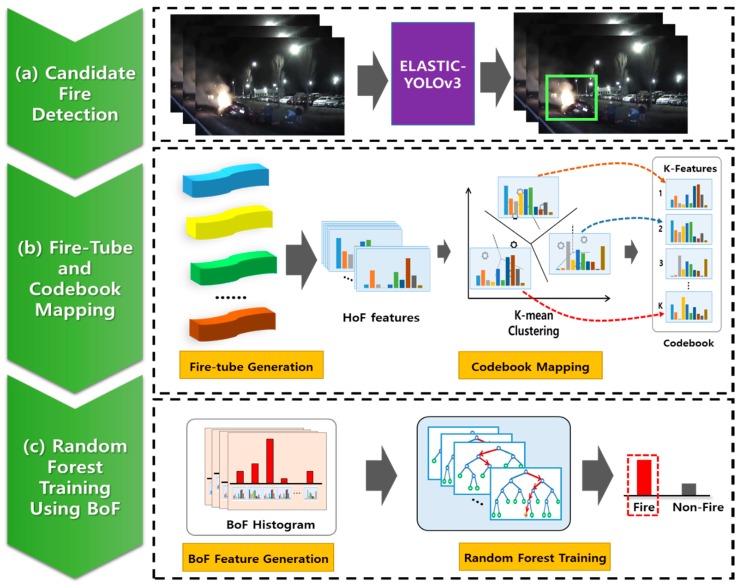
Overview of the proposed method for night-time fire detection: (**a**) fire candidate regions are extracted from consecutive images using ELASTIC-YOLOv3, and (**b**) a fire-tube is constructed by connecting the fire candidate areas, where the HoF are generated by the orientation of the motion vectors for estimating the temporal information of a fire-tube. Each HoF is transformed into a BoF histogram through codebook mapping. (**c**) The BoF are generated from fire and fire-like regions, and a random forest (RF) classifier is trained.

Figure 2 shows the overall structure of the proposed framework. To detect night-time fires having irregular movements within a short time effectively, this study first detects the candidate fire areas using a lightweight CNN (Figure 2a). We combine an ELASTIC block with the YOLOv3 network [36] to improve the detection rate for small objects when considering the processing time and accuracy. To obtain the temporal information in the detected fire candidate areas, we construct a fire-tube by connecting the fire candidate areas that appear in consecutive frames. From the fire-tubes, the motion orientation between frames is estimated by an optical flow, and a histogram of oriented features (HoF) is generated based on the orientation of the motion vectors. Each HOF is transformed into a bag-of-features (BoF) histogram through codebook mapping (Figure 2b), and the final verification of the fire area is achieved using a random forest (RF) classifier (Figure 2c), which has a fast processing time and high accuracy with the transformed feature vectors.

The remainder of this paper is structured as follows. We present an overview of the related studies on camera-based fire detection in Section 2. Section 3 provides the details of our proposed method in terms of the feature extraction and classifier. Section 4 provides a comprehensive evaluation of the proposed method through various experiments. Finally, some concluding remarks are given in Section 5.

## 3. Fire Detection Methods Using ELASTIC-YOLOv3 and Fire-Tube Analysis

### 3.1. ELASTIC-YOLOv3

The most important factor in fire detection is the early-stage detection starting from a very small sized fire. Among the several object detection algorithms used for detecting early fire candidate areas, we adopted fast YOLOv3 [36], which is an upgraded version of YOLO [37]. YOLOv3 [36] has the advantage of being able to operate on both a CPU and a GPU because the object detection speed is extremely fast. However, it has a disadvantage in that the detection performance of small objects is low compared to that of Faster R-CNN [31] or single-shot detector (SSD) [38].

Recently, ELASTIC Block [39] was introduced to improve the detection performance of objects of various sizes by applying downsampling and upsampling in the convolution layer while keeping the number of parameters similar to the computations of the existing CNN model. Therefore, we propose the use of an ELASTIC-YOLOv3 network by combining an ELASTIC block with the existing YOLOv3 network to improve the detection accuracy in a small sized fire region.

**Figure 3:**
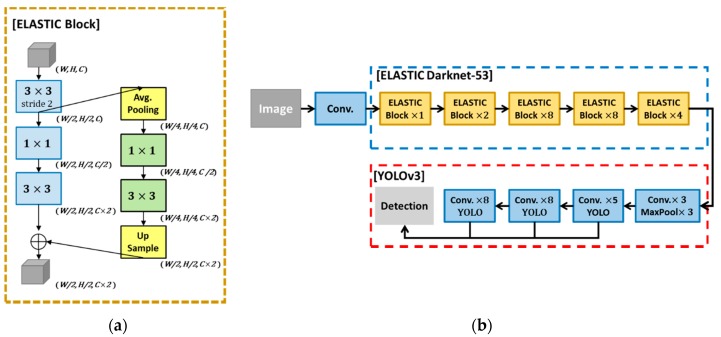
ELASTIC-YOLOv3 structure with proposed ELASTIC block. (**a**) The elastic block was upsampled from the average pooled input along Path 2 through two convolutions at low resolution and concatenated back to the original resolution, and (**b**) an ELASTIC-YOLOv3 structure with an ELASTIC block was coupled to Darknet-53, which is YOLOv3’s base network. The symbol × indicates the number of repetitions of the block.

The ELASTIC block is divided into Path 1 (blue boxes), in which three convolutions are applied, and Path 2, in which upsampling is conducted after an average pooling and continuous 1 × 1 and 3 × 3 convolutions, as shown in Figure 3a. Unlike the initial ELASTIC block, modified ELASTIC applies a 3 × 3 convolution with a stride of 2 to the input feature maps to reduce the size by half and applies them to the input of each path. Instead of downsampling in Path 2, average pooling is applied to minimize the loss of feature information. Because the ELASTIC block can extract features that reflect various sizes of objects, compared to a conventional CNN through two paths, it can increase the detection rate for both small and large objects. In addition, because the ELASTIC block divides the existing single path into two paths, the number of parameters and operations can be maintained similar to the original single path.

An ELASTIC block was applied to Darknet-53, the base network of YOLOv3, as shown in Figure 3b. As indicated in the figure, ELASTIC Darknet-53 (blue dotted line) consists of five consecutive blocks, and each block is executed repeatedly between one and eight times. The YOLOv3 part (red dotted line) uses the same structure as YOLOv3.

We used ELASTIC-YOLOv3 to detect candidate fire regions for the input images. The fire detection performance of ELASTIC-YOLOv3 is demonstrated in Section 4.

### 3.2. Temporal Fire-Tube Generation Using the Histogram of Optical Flow

**Figure 4:**
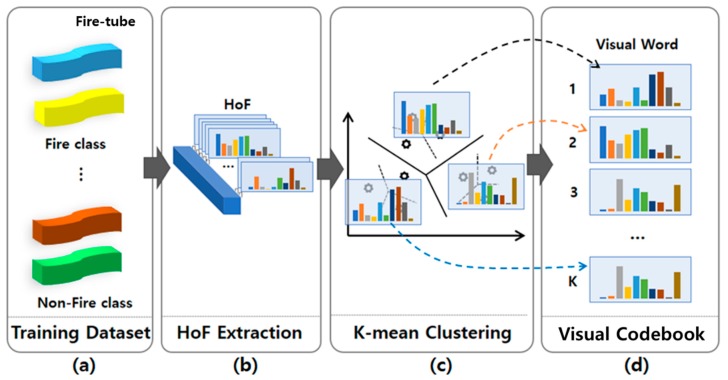
Codebook generation process. (**a**) Fire-tube generated fire and non-fire training classes; (**b**) HoF extraction from the fire-tube of the training dataset; (**c**) K-means clustering; and (**d**) visual codebook generation.

Because fire flames constantly vary in terms of shape and irregularity depending on the burning material and wind conditions, it is necessary to observe multiple frames to consider the above characteristics over time. In a similar manner to that described in Ko et al. [40], we first generate a 3D fire-tube by combining the candidate blocks with N corresponding blocks in the previous frames, as shown in Figure 4a. Each tube has a different width and height ($\Delta_{w}$,$\Delta_{h}$) and the same time duration $\Delta_{n}$. In a previous study [35], the width and height of the tube were determined to be the same as the size of the fire region of the current frame, whereas in this study, the size and location of the fire zone are changed according to the fire information of each frame.

In addition to the above-mentioned factors, there is a difference in the movement of the fire flame depending on the distance between the camera and burning point. Owing to the distance between the camera and fire flame and the irregular movement of the fire, an effective way to reduce false detections is by stacking only those frames in which a change occurs in the fire-tube rather than every frame. To solve this problem, every frame goes through a skip-frame process to determine the frames to stack. If the index of the currently detected frame is *i*, the fire region of the current *i*th frame is stacked into the first region of the fire-tube (*k* = 0). Next, moving backward through the video sequences, the magnitude of the HoF between the fire region of the (*i*−1)th frame and *k*th region of the fire-tube is compared. If the difference in magnitude between two frames is greater than a threshold (*th*), the (*i*−1)th frame is stacked on the fire-tube and the fire-tube index (k) is increased. Otherwise, the (*i*−1)th frame is skipped and not stacked on the fire-tube. This process is repeated until the fire-tube is full (N). Here, N was set to 50, and the threshold *th* was adjustable according to the distance between the camera and monitored object. In addition, the maximum number of skip frames was limited to three.

The fire-tube generation process when considering the motion variation of the fire is shown in Algorithm 1.
**Algorithm 1** Skip Frame for Fire-Tube GenerationF-Tube: A set of fire-tubes Initialize F-Tube=∅, **N:** size of **F-Tube**$HoF_{mag}$: Magnitude of HoF *th*: a threshold for skip frame selectionk: index of fire-tube, i: index of current candidate frame (i > N (50))K = 0, F-Tube[k]=frame (i)
i--**Do**   $\mathrm{If}\;\mathrm{frame}\;\left(i\right)==fire\;\&\;\left|F\text{-}Tube\left[k\right].{\mathrm{HoF}}_{\mathrm{mag}}-\mathrm{HoF}{\left(i\right)}_{\mathrm{mag}}\right|\geq th$   Then   **F-Tube** [k] = frame (i)   k++, i--   Else     i--//skip frame**While** (No. of frames of **F-Tube** is full)

We compute the HoF from each fire-tube, as shown in Figure 4b. Because the size of the fire regions belonging to the fire-tube varies, the HoF of the same dimension is extracted by applying the spatial pyramid pooling (SPP) [41] from each fire-tube region. Each HoF is discretized into nine orientations including zero motion, and each discrete orientation is binned according to the magnitude. Because SPP partitions the region into divisions from finer to coarser levels and aggregates local features into a single feature, it does not require normalizing the region beforehand and is not affected by the scale or aspect ratio of the region. We apply two-level spatial pyramids {1 × 1 and 2 × 2} to extract a nine-dimensional HoF from each divided region and concatenate the extracted HoFs to obtain 45-dimensional combined HoF. Therefore, the feature vector created in one fire-tube becomes 2205 dimensions throughout the following equation:

(1) $$\mathrm{Concat}\_\mathrm{HoF}=\overset{L}{\underset{l=1}{\sum }}l^{2}\times \left(N-1\right)\times \dim\left(\mathrm{HoF}\right)$$

HoF consists of nine dimensions (dim), and *L* indicates the SPP level. A total number of five blocks are generated in a single frame by applying the SPP (including a 1 × 1 global block).

### 3.3. Bag-of-Features Extraction from HoF and Fire Verification

In this section, we describe how to extract feature vectors in 2205 dimensions for a fire-tube. If multiple fire candidates are detected in a video, it takes considerable time to extract the feature vectors from multiple fire-tubes at the same time. Therefore, we use the BoF to shorten this feature vector extraction.

To apply the BoF, we first create a visual codebook consisting of 35 visual words (clusters) through K-means clustering in the training dataset, as shown in Figure 4. The visual codebook consists of 30 visual words for the fire class and 5 visual words for the non-fire class. Once the visual codebook of the BoF is built, two types of BoF histograms should be estimated for the fire and non-fire classes. The BoF histogram assigns each feature to the closest visual word and computes the histogram of visual word occurrences over a temporal volume [41]. The K-dimensional BoF histogram is estimated from the visual codebook by means of binary weighting, which indicates the presence or absence of a visual word based on values 1 and zero, respectively. All weighting schemes apply a nearest-neighbor search in the visual codebook, in the sense that each fire-tube is mapped to the most similar visual word. The reduced feature vector is finally determined based on the last random forest classifier, regardless of whether the fire is real.

Although the performance of CNN has become superior to that of existing machine learning (ML)-based methods in terms of image classification problems, CNN requires a large number of hyperparameters, such as the learning speed, cost function, normalization, and mini-batch, as well as careful parameter adjustments. Therefore, it cannot be effectively generalized when the training data are small in number [42]. By contrast, RF, which is a representative ML algorithm, has a relatively lower performance than CNN for high-dimensional and continuous data such as image and audio data. However, when the data are of a tabular type and the number of training data is small, the RF can be quickly learned and tested with fewer parameters [43]. In this study, although the input data were made up of image sequences, we used an RF instead of a CNN or an RNN because BoF is tabular data, the number of training data are limited, and a fast fire detection was needed.

**Figure 5:**
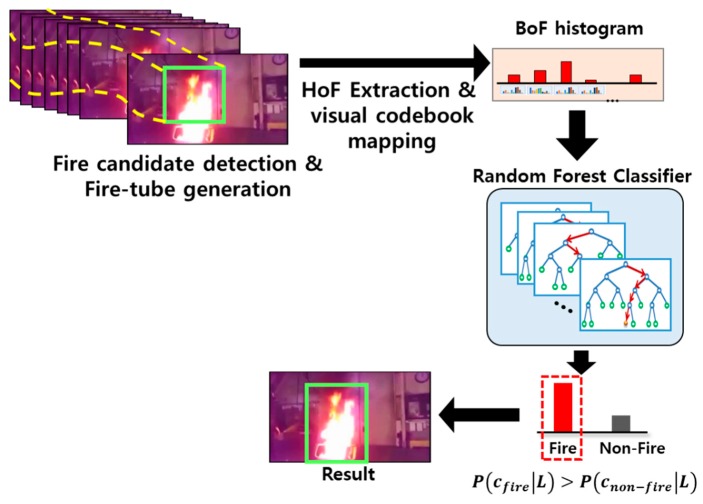
Fire verification procedure at test time. From the fire-tube, the BoF is generated through the same training and is input to the RF for final fire verification.

During the RF learning process, referring to the experiment results of Ko et al. [44], the maximum depth of the tree was set to 20, and the number of trees constituting the RF was set to 120. Once the RF was trained, the BoF histogram of the test fire-tube was generated and distributed into the trained RF. Each feature corresponded to one leaf of a decision tree in the RF. To compute the final class distribution, we arithmetically averaged the probabilities of all trees, $L=\left(l_{1},l_{2},\ldots ,l_{T}\right)$, as follows:

(2) $$P\left(c_{i}|L\right)=\frac{1}{T}\sum_{t=1}^{T}P\left(c_{i}|l_{t}\right),\;\;i\epsilon \left\{fire,\;non-fire\right\}$$

where T is the number of trees. We chose $c_{i}$ as the final class of an input image if $P\left(c_{i}|L\right)$ had the maximum value. Figure 5 describes the detailed procedure of the testing based on the BoF of the fire-tube and RF.

## 4. Experimental Results

The benchmark dataset associated with camera-based fire detection is relatively small compared to other research areas. Vision based fire detection(VisFire) [45] and the Keimyung University (KMU) Fire & Smoke Database [46], two known databases related to fire detection, were unsuitable for the purposes of this study because they are mostly for daytime and nearby fire videos. Therefore, we constructed a night-time fire dataset in this study (NightFire-DB), which included some night-time videos from the KMU Database [46] and new night-time fires downloaded from YouTube. NightFire-DB was a collection of various night-time fire and fire-like videos in an urban environment including roads, large factories, warehouses, shopping malls, parks, and gas stations taken at night. There were a total of 14 fire and 10 non-fire videos for training. The test videos consisted of 10 fire videos and 10 non-fire videos. The average number of frames of each video was 1030 frames, and the frame rate was 30 Hz, whereas the size of each input video varied from a minimum pixel resolution of 320 × 240 to a maximum pixel resolution of 640 × 480. Table 1 describes the detailed configuration of NightFire-DB.

The dataset for training the proposed ELASTIC-YOLOv3 consisted of still images of night-time fires from NightFire-DB. This dataset consisted of 4000 static images of night-time fire and fire-like images. In addition, for learning about the proposed temporal fire-tube-based fire verification method, 600 fire-tubes were randomly selected from NightFire-DB: 300 fire-tubes with fire and 300 fire-tubes without fire in the presence of tenuous fire-like lights. We measured the precision, recall, and F1-score to evaluate the performance of the proposed algorithm.

To determine fire detection, we declared that a fire was detected correctly when the overlap rate between the actual ground truth area and the detected area was more than 50%. We used the popular precision, recall and F1-score (the harmonic mean of precision and recall) as the evaluation measures for the fire and object detection:

(3) $$\mathrm{Precision}=\frac{TP}{TP+FP}$$

(4) $$\mathrm{Recall}=\frac{TP}{TP+FN}$$

(5) $$F1-\mathrm{score}=2\times \frac{1}{\frac{1}{\mathrm{Precision}}+\frac{1}{\mathrm{Recall}}}=2\times \frac{\mathrm{Precision}\times \mathrm{Recall}}{\mathrm{Precision}+\mathrm{Recall}}$$

The system environment for the experiment included Microsoft Windows 10 and an Intel Core i7 processor with 8 GB of RAM. The proposed ELSATIC-YOLOv3 operated based on a single Titan-XP GPU, and the RF algorithm was tested using a CPU.

### 4.1. Performance Evaluation of Fire Candidate Detection

The number of codebooks that determined the size of the BoF was an important factor in extracting accurate temporal characteristics from the fire-tube. A smaller sized codebook could reduce the number of dimensions of the BoF, although in this case, such a codebook may lack classification power because HoF with different properties could be assigned within the same cluster. By contrast, a larger sized codebook could reflect various HoF properties, but was sensitive to noise and required additional processing time [40].

**Figure 6:**
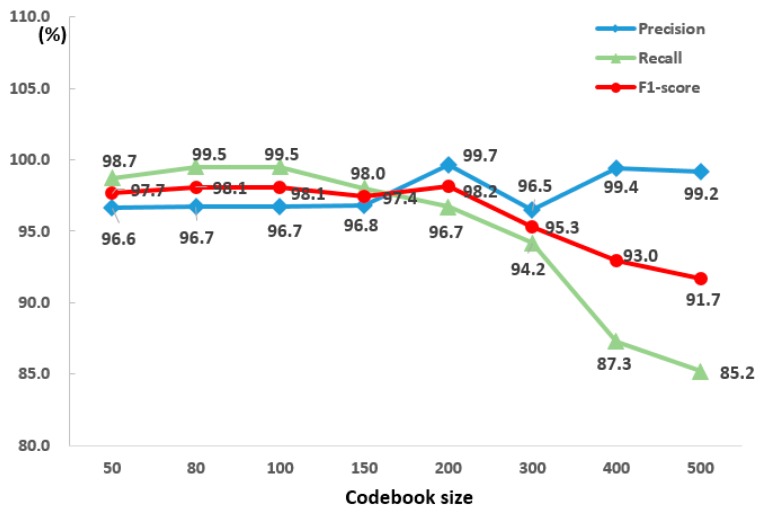
Nine pairs of experimental results to determine the codebook size.

To determine the appropriate number of codebook sizes, this study compared the precision and recall by varying the sizes of codebooks for some of the test data (real-fire01, 03, 05, 06, 07 and 08). As shown in Figure 6, as the size of the codebook increased, the two measures increased simultaneously. However, when the size reached over 150, the precision was maintained, but the recall reduced steeply. If the codebook size was 150 or more, the number of false positives decreased, and the precision was maintained, whereas the number of false negatives increased rapidly, and the recall continued to reduce. This was because as the codebook grew in size, fewer fire-tubes were allocated to the outlier cluster. However, in a real fire, it is more dangerous to miss a fire (false negative) than to experience a false detection (false positive). Therefore, in this study, the most appropriate codebook size was 80 when considering the accuracy and computational time for generating a codebook.

### 4.2. Performance Evaluation of Fire Candidate Detection

To validate whether the proposed ELASTIC-YOLOv3 for the detection of a fire candidate could be effectively applied to test the NightFire-DB dataset, a performance evaluation was conducted based on the difficulties of the dataset defined into three levels according to their size: small, medium, and large. A small fire was defined as a case in which the fire size was less than 10% of the size of the original input frame, a medium fire as less than 30%, and a large more than 30%. For the performance evaluation, we first compared the precision, recall and F1-score against the original YOLOv3 [36].

As shown in Table 2, in terms of the precision, YOLOv3 and ELASTIC-YOLOv3 showed similar detection performances regardless of the fire size. However, in terms of the recall, the proposed ELASTIC-YOLOv3 improved by 10.2% for small fires and 6.1% for medium sized fires compared with YOLOv3. The relatively higher recall rate of the ELASTIC-YOLOv3 method meant that the proposed method detected fire candidates well without false negatives (missing fires) even in small sized fire regions. Therefore, the proposed ELASTIC-YOLOv3 showed a better performance than YOLOv3 even for small sized fires. In terms of the F1-score, ELASTIC-YOLOv3 achieved good even scores across all fire sizes, whereas YOLOv3 had a large gap in score between large and small sized fire regions.

In addition, we compared the fire candidate detection performance and processing time per frame with the Faster R-CNN- [33] and single-shot detector (SSD)-based fire detectors, which are widely used in object detection and other fire detection studies. Table 3 shows the comparison results of the four methods. Note that the SSD showed the highest precision, whereas the recall rate was the lowest at 67.7%. Owing to an imbalance of the precision and recall, the F1-score was 80.4, which was 17% lower than ELASTIC-YOLOv3. The Faster R-CNN showed a better fire detection performance than that of the SSD, but required twice as much processing time. YOLOv3, as is well known, has a much faster processing time than SSD and Faster R-CNN. It was also found in the experimental results that its fire detection performance was superior to those of the other two methods even for various sizes of fires. The proposed ELASTIC-YOLOv3 required 0.1 ms more processing time than the original YOLOv3, although the recall and F1-score were largely improved.

### 4.3. Performance Evaluation of Temporal Fire-Tube and BoF

In this section, we evaluate the performance of a fire verification algorithm to determine whether it correctly reflects the irregular movement of a fire using the BoF based on a fire-tube and RF classifier. Because the proposed algorithm (ELASTIC-YOLOv3 + RF) should be compared to similar algorithms that reflect the dynamic nature of a fire region, we first applied ELASTIC-YOLOv3 and input the fire candidate regions into a deep three-dimensional convolutional network (3D ConvNets) [48] algorithm and LSTM. 3D ConvNets [48] is a popular deep learning algorithm for training spatiotemporal features for a video analysis, such as action recognition, abnormal event detection, and activity understanding. Because action recognition requires a spatiotemporal feature analysis similar to fire, 3D ConvNets are combined with ELASTIC-YOLOv3 (ELASTIC-YOLOv3 + 3D ConvNets [48]). In the case of LSTM, we adopted the method in [33] to apply a feature vector converted from a CNN to the LSTM layer in a frame-by-frame manner and verify the fire through a continuous LSTM output. Like other comparison methods, only frames detected by ELSATIC-YOLOv3 as fire candidates were input into the LSTM (ELASTIC-YOLOv3 + LSTM [33]). All experiments were measured after 50 frames because fire verification was required only after a certain frame had been accumulated. With the proposed method, a GPU was used for ELSATIC-YOLOv3, and a CPU was used simultaneously for the RF.

Because all three comparison methods were a type of post-processing based on the fire candidate detection results of ELASTIC-YOLOv3, there were two factors regarding the point noted in Table 4. The first was how well the algorithms removed false positives that were detected incorrectly, and the second (by contrast) was how well the algorithms maintained true positives that were detected correctly.

As shown in Table 4, the performance of the proposed method was superior to that of the other methods [33,48] in terms of the recall, F1-score, and processing time. In particular, the recall rates of two of the comparison methods were much lower than that of the pre-processing of ELASTIC-YOLOv3, whereas the proposed method showed an improvement of 2.6%. This meant that the proposed fire-tube method applying an RF reflected the dynamic nature of a flame well. Moreover, a better recall rate is a more important factor in evaluating the performance, because it is more dangerous to miss a fire than to have a false detection. In terms of precision, the ELASTIC-YOLOv3 + LSTM [33] method was approximately 21% lower than the pre-processing, whereas the proposed method had a 1.8% decrease over the pre-processing. This meant that the precision of the ELASTIC-YOLOv3 + LSTM [33] method was degraded because the true positives and false positives were filtered as a non-fire, whereas the proposed method could effectively verify the fire candidates detected during the pre-processing using the fire-tube and RF classifier. In addition, because the proposed post-processing method simply extracted the BoF from the fire-tube and verified the fire using an RF when applying both the GPU and CPU at the same time, the processing time was faster than the other two methods using only a GPU.

**Figure 7:**
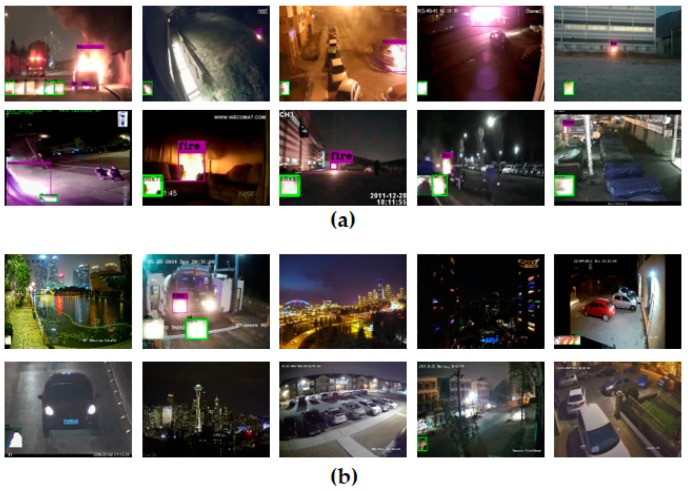
Fire detection results using NightFire-DB: (**a**) ten real fire videos and (**b**) ten non-fire videos. The green boxes at the bottom of the images are the candidate fire regions when using ELASTIC-YOLOv3, and the red boxes are the final results of the proposed RF when applying the fire-tube method.

Figure 7 shows the fire detection results obtained using our proposed method and the NightFire-DB test data. We see that the proposed algorithm could detect a real fire correctly even with other lights around the fire, such as streetlights, neon signs, and car headlights, and could detect some small fires at a long distance. However, the proposed algorithm occasionally incurred a false detection in a non-fire region (e.g., the second image in Figure 7b) if a change in size change continuously occurred, such as when the car headlights moved forward in front of the camera.

## 5. Conclusions

In this paper, we presented a new night-time fire detection method in an urban environment based on ELASTIC-YOLOv3 and a temporal fire-tube. As the first step of the algorithm, we proposed the use of ELASTIC-YOLOv3, which can improve the detection performance without increasing the number of parameters through improvements to YOLOv3, which is limited to the detection of small objects. For the second step, we proposed a method for generating a dynamic fire-tube according to the characteristics of the flame, a method for extracting the movement of the flame using an HoF, and an algorithm for converting the HoF into the BoF. Fire candidate regions detected using ELASTIC-YOLOv3 were quickly validated using the HoF and BoF extracted from the fire-tube and RF classifiers. The experimental results showed that the proposed method using both a GPU and CPU was faster than deep learning and LSTM-based state-of-the-art fire detection approaches. Based on the experimental results, we see that the proposed method could be successfully used for real-time fire detection at night in urban areas owing to the high accuracy and fast processing speed. Because the proposed method in this paper was an experiment on various fire videos collected on YouTube, the results may vary depending on changes in the surrounding environment, changes in weather, and camera conditions when applied in a real urban environment. Therefore, it is necessary to collect more databases and analyze the results assuming various urban environments in the future.

The current algorithm required the use of a GPU for ELASTIC-YOLOv3; hence, there was a limit when applying it to low-end embedded systems. However, if YOLOv3 were upgraded to a tiny version that could run on a CPU, it could be optimized for embedded systems. Moreover, for future research, we plan to improve the algorithm to detect forest fires at night using a long-distance camera (installed at a monitoring tower) in addition to nearby night-time flame detection by developing new types of fire-tube and BoF algorithms.

## Figures and Tables

**Table 1:** Configuration of test video sequences including fire and non-fire videos.

Fire Video No.	Total No. of Frames	Non-Fire No.	Total No. of Frames
real-fire01	1788	non-fire01	343
real-fire02	862	non-fire02	1439
real-fire03	1187	non-fire03	1388
real-fire04	1131	non-fire04	913
real-fire05	683	non-fire05	575
real-fire06	1121	non-fire06	514
real-fire07	1787	non-fire07	1269
real-fire08	1786	non-fire08	686
real-fire09	1260	non-fire09	665
real-fire10	718	non-fire10	473
Average	1232	Average	827

**Table 2:** Comparison of the precision, recall, and F1-score according to the size of fire regions for the proposed ELASTIC-YOLOv3 and original YOLOv3 [19] using the NightFire-DB.

Methods		Precision (%)	Recall (%)	F1-Score (%)
YOLOv3 [36]	Small	97.5	81.9	89.0
	Medium	98.8	92.6	95.6
	Large	97.6	99.2	98.4
	Average	98.4	90.3	94.2
Proposed ELASTIC-YOLOv3	Small	96.3	92.1	94.1
	Medium	99.8	98.7	99.3
	Large	99.4	100	99.7
	Average	98.8	97.0	97.9

**Table 3:** Comparison of the precision, recall, F1-score, and processing time of four methods using NightFire-DB. SSD, single-shot detector.

Methods	Precision (%)	Recall (%)	F1-Score (%)	Processing Time (ms)
SSD [38]	99.0	67.7	80.4	22.5
Faster R-CNN [47]	81.7	94.5	87.2	45
YOLOv3 [36]	97.9	91.2	94.3	15.9
ELASTIC-YOLOv3	98.5	96.9	97.7	16

**Table 4:** Comparison of the precision, recall, F1-score, and processing time of four comparison methods using the NightFire-DB. The processing time of each algorithm included 15 ms of ELASTIC-YOLOv3.

Methods	Precision (%)	Recall (%)	F1-Score (%)	Processing Time (ms)	Operation
ELASTIC-YOLOv3 + 3D ConvNets [48]	97.9	89.3	89.3	20.4	GPU
ELASTIC-YOLOv3 + LSTM [33]	76.7	99.5	86.6	56	GPU
ELASTIC-YOLOv3 + RF classifier	96.7	99.5	98.0	24.8	GPU (YOLOv3) + CPU (RF)
